# Intravenous Dexamethasone Reduces Pain in Middle Ear Surgery

**DOI:** 10.22038/IJORL.2022.60292.3077

**Published:** 2022-11

**Authors:** Imen Zouche, Feris Abdelmalak, Zied Triki, Salma Ketata, Moncef Sellami, Abdelhamid Karoui

**Affiliations:** 1 *Department of Anesthesiology, Habib Bourguiba University Hospital,* *Faculty of Medicine, University of Sfax, 3000 Sfax, Tunisia.*; 2 *Department of Otorhinolaryngology, Habib Bourguiba University Hospital, * *Faculty of Medicine, University of Sfax* *, 3000 Sfax, Tunisia.*

**Keywords:** Analgesia, Dexamethasone, Middle ear, Postoperative pain

## Abstract

**Introduction:**

Few studies evaluated the treatment of postoperative pain in middle ear surgery.

**Materials and Methods:**

We conducted a randomized clinical trial to evaluate the efficacy of dexamethasone in the management of postoperative pain in middle ear surgery. Group G1 received an intravenous injection of 2 ml of physiological saline 30 minutes before the end of the procedure. Group G2 received a 2 ml intravenous solution containing 8 mg of dexamethasone, 30 minutes before the end of the procedure. Pain perception was measured by the Visual analog scale (VAS) every 10 min during the first hour and then every 6 hours during the 24 hours postoperatively. The delay of the first analgesic demand and the consumption of analgesics use during the first 24 hours postoperatively, were recorded.

**Results:**

VAS values were lower in G2at all measurement points during the first hour, as well as the first 24h postoperatively (Mann-Whitney test, P<0.05).The delay of the analgesic request was (0 (0-60) for G1 versus 0 (0-80) for G2, P=0.04, Mann-Whitney test). Morphine was used in 44% of the patients in G1 against 19% for G2 (P = 0.031). There was a significant difference between G1 and G2 in terms of the total dose of morphine consumed (P= 0.028, Mann-Whitney test). Paracetamol demand was lower in group 2 at all points of assessment during the first 24 hours postoperatively.

**Conclusions:**

Intravenous dexamethasone is effective in decreasing pain and analgesic requirement, during the first 24 hours postoperatively, in patients undergoing middle ear surgery.

## Introduction

Middle ear surgery is associated with moderate postoperative pain (1). Many protocols have been used to manage post-operative pain in numerous surgeries. Dexamethasone was involved in many of these protocols (2–6) and showed analgesic efficacy and fewer side effects than other drugs, mainly opioids (7).

However, few studies have evaluated the treatment of postoperative pain in middle ear surgery (3, 8). We aimed to evaluate the impact of intravenous dexamethasone on postoperative pain and analgesic requirements after middle ear surgery.

## Materials and Methods

Following the approval of the Ethics Committee (no 16/ 2016), we conducted a randomized double-blind clinical trial, conducted within the anesthesia-intensive care unit and the otorhinolaryngology department. 

***Patient selection***

All the patients who were proposed for middle ear surgery during the study period, aged 20 to 65 years and classified according to the classification of the American Society of Anaesthesiologists (ASA) ASA I or II, were included in our study. 

Patients with unstable diabetes, or contraindications to morphine or paracetamol, as well as patients on long-term corticosteroid therapy, those who had used opioids analgesia or nonsteroidal anti-inflammatory drugs in the 2 days before surgery, and pregnant women, were not included in this study. Patients who did not follow the study protocol and those who had an intraoperative complication (anesthetic or surgical complication) or whose duration of surgery exceeded 3 hours were excluded from the study. 

***Patient*** ***consent***

Patients were informed of the analgesic protocol and the use of the visual analog scale (VAS). Signed consent was essential for inclusion in the study.

***Randomization of groups***

The patients proposed for middle ear surgery who were included in our study were randomized into two groups using Research Randomizer Version 4.0software: Group G1: received an intravenous injection of a 2 ml solution of physiological saline 30 minutes before the end of the procedure.

Group G2 received a 2 ml intravenous solution containing 8 mg of dexamethasone, 30 minutes before the end of the procedure.

For both groups, an anesthesiologist not participating in the study prepared the solutions to be injected with equal volumes into unlabeled syringes.

***Protocol of the study***

The electrocardiogram (ECG), non-invasive blood pressure (NIBP), and pulse oxygen saturation were all used to monitor every patient (SpO2).Vascular filling with 10 ml/kg of 0.9% isotonic saline began after a 20-gauge venous needle was inserted.

For all patients, the general anesthetic regimen was standardized. Propofol 3 mg/kg, fentanyl 3 g/kg, and cisatracurium 0.15 mg/kg were slowly injected intravenously throughout induction. Patients were intubated using a customized tube after three minutes of pure oxygen breathing with a facial mask. Isoflurane with a minimum alveolar concentration (MAC) of 1%, fentanyl at a dose of 0.5 mg per kilogram every time the heart rate (HR) or systolic blood pressure (SBP) changes by more than 20% from baseline values, and cisatracurium at a dose of 0.02 mg per kilogram every 30 minutes were used to maintain anesthesia. 

The pressure of exhaled CO2 (PETCO2) was observed. Using a controlled volume mode and a tidal volume of 8 ml/kg, artificial ventilation was given. To achieve a PETCO2 between 30 and 35 mmHg, a respiratory rate that was begun at 12 cycles/min and customized for each patient was used. An equimolar mixture of 50% air and 50% oxygen was supplied, with an inspiration/expiration ratio fixed at 12. In the operating room, the patients were all extubated. 

All of the patients were sent to the post-anesthetic care unit (PACU) for two hours following extubation. All of the patients were kept at the department of oto-rhino-laryngology for at least 24 hours following surgery. A medical professional who was blinded to the group the patient was assigned to collect the intraoperative and postoperative parameters.

***Analgesia protocol***

15 minutes before the end of the procedure, patients were given 1g of paracetamol intravenously. Following surgery, supplementary analgesia was given by:

-A titration of morphine (1 mg intravenously, every 5 minutes) if the value of the visual analog pain scale (VAS)> 3 in the PACU.

-1 g of paracetamol was given intravenously every six hours at the otorhinolaryngology department whenever VAS> 3. 

***Criteria for judgment***

The usage of non-opioid analgesics in the first 24 hours postoperatively and VAS pain at that time were the two main objectives of our study.

***Statistical analysis***

To achieve a difference in the immediate postoperative pain scores of 20mm (standard deviation of 25mm), with a power of 0.8 and =0.05, each group needed to have at least 25 patients. We consequently chose to enroll 27 patients per group. Then entered data into SPSS version 23 for Windows, and we conducted a statistical analysis. A number with a percentage was used to represent qualitative variables, on the average standard deviation for continuous variables that followed a normal distribution, and a median distribution with extremes for continuous variables that did not follow a normal distribution. For the examination of qualitative variables, the chi-square test (also known as Fisher's exact test) was applied. For the study of the quantitative variables, the Mann-Whitney test or Student's t-test was applied when the variable was followed by a normal distribution. The Kolmogorov-Smirnov test was used to determine whether all continuous variables followed a normal distribution. P<0.05 was considered a significant level.

## Results

***Study Population***

 The number of subjects selected was 54, divided into two groups of 27 each. Patients, whose operation duration exceeded 3 hours in length, were excluded from the study (two from each group). The study took place over 18 months (April 2017 to October 2017).

Demographic parameters and pre-operative anesthetic parameters were statistically comparable between the two groups (Table 1).

Intraoperative parameters were statically similar in both groups (Table 2).

**Table1:** Comparison of demographic parameters and pre-operative data between the 2 groups

	**Group G1 (N = 25)**	**Group G2 (N = 25)**	** p**
Age( Year) ±SD	43.9 ±12.6	39.3 ±15.3	0.253*
Gender (M/W)	7/18	15/10	0.045
Weight (Kg)	70 (55-94)	70 (60-100)	0.39†
ASA (I/II)	19/6	23/2	0.123

SD: standard deviation; M: man; W: Woman; ( ): extremes; *: student test; †: Mann-Whitney test. Kg: kilogram

**Table 2:** Comparison of chirurgical parameters

	**Group G1 (N = 25)**	**Group G2 (N = 25)**	** p**
Duration of surgery (min)±SD	102.20 ±26.42	113.80 ±25.30	0.119*
Duration of anesthesia (min)±SD	140±65	150±53	0.187*
Average consumption of fentanyl(gamma)	350 (200-600)	400 (200-500)	0.92†

SD: standard deviation**; ( ):** Extremes; *: student test; **†**: U Mann-Whitney test; N: Number

***Assessment of postoperative pain***

A comparison of the VAS values ​​between the two groups (Table 3) showed a significant difference at all the measurement points during the first hour postoperatively (Mann-Whitney test, P<0.05). 

**Table 3:** VAS comparison during the first post-operative hour between the two groups

	**Group 1**	**Group 2**	**Signification (p)**
VAS 0 min	2 (0-8)	0(0-8)	0.001†
VAS 10 min	2(0-7)	0(0-8)	0.008†
VAS 20 min	1(0-7)	0(0-8)	0.009†
VAS 30 min	2(0-6)	0(0-10)	0.002†
VAS 40min	2(0-7)	0(0-10)	0.002†
VAS 50 min	2(0-7)	0(0-5)	0.002†
VAS 60 min	2(0-4)	0(0-4)	0.00 3†

VAS: visual analog pain scale; ( ): Extremes; †: U test of Mann-Whitney

The comparison of the VAS values ​​during the first 24 hours of hospitalization between the two groups (Table 4) showed a significant difference at all measurement points (Mann-Whitney test, P <0, 05).

**Table 4:** VAS comparison during the first 24 hours post-operatively between the two groups

	**Group 1**	**Group 2**	**Signification (p)**
VAS H6	5(0-8)	3(0-8)	0.010†
VAS H12	4(0-8)	1(0-6)	0.006†
VAS H18	3(0-8)	1(0-5)	0.001†
VAS H24	3(0-8)	0(0-4)	0.000†

( ): extremes; †: U Mann Whitney test; VAS: visual analog pain scale

The delay of the analgesic request was statistically greater in group 2 than that in group 1 (0 (0-60) for group 1 versus 0 (0-80) for group 2, p = 0.04, Mann-Whitney test).

The use of morphine was more frequent in patients in group 1. Forty-four percent of the patients in G1 required the use of morphine titration against 19% for G2 (P= 0.031). There was a significant difference between the total dose of morphine consumed by the patients in the two groups (P= 0.028, Mann-Whitney test).

Paracetamol demand was lower in group 2 at all points of pain assessment during the first 24 hours postoperatively (Table 5).

**Table 5:** comparison of the need for paracetamol during the first 24 postoperative hours between the two groups

**Need of paracetamol**	**Group1**	**Group 2**	**Signification (p)**
H6	80%	40%	0.004^¥^
H12	76%	32%	0.002^¥^
H18	56%	20%	0.009^¥^
H24	44%	16%	0.0031^¥^

H: Hour; ^¥^: Chi 2 test

## Discussion

In our study, the use of dexamethasone was effective in reducing pain during the first postoperative hour, as well as during the first 24 hours. The delay of the first request for analgesia was longer forG2, the group receiving dexamethasone. 

Mechanism of action of dexamethasone on pain: Dexamethasone acts according to two different mechanisms:

Glucocorticoids cause, in a few seconds to a few minutes, a reduction of neuronal discharges. This specific action is not linked to protein synthesis (9, 10). Nuclear factor kappa-B (NF-B) activity is decreased and anti-inflammatory cytokines are produced when glucocorticoids are present (11).

Glucocorticoids have a delayed anti-inflammatory action that requires protein synthesis. They decrease the synthesis of prostaglandins in the peripheral tissues and the central nervous system. Additionally, the production of a microsomal prostaglandin synthetase involved in pain sensitivity is inhibited by glucocorticoids. Preoperative administration would be advised to take advantage of the anti-inflammatory activity of glucocorticoids given this delayed effect (12,13). Dexamethasone doses over 0.1 mg/Kg lower pain at the 12th and 24th postoperative hours and reduce morphine consumption, according to a 2011 meta-analysis that looked at the impact of a single injection of the medication on postoperative pain across all procedures (14). AHN et al (15) evaluated the efficacy of dexamethasone in reducing postoperative nausea and vomiting, and pain after middle ear surgery. The authors did not conclude a significant decrease in VAS at the 3^rd^, 6^th^, and the 24^th^post-operative hours. They explained the lack of analgesic effect to the degree of inflammation and concluded that dexamethasone cannot reduce the pain associated with significant surgical trauma, but can relieve mild pain.

 In the meta-analysis of Waldron et al (7), patients receiving dexamethasone had a longer delay of the first analgesic request. In our study, 44% of the patients in the saline group required morphine titration versus 19% for the dexamethasone 8 mg group during the PACU stay. A study of posterior lumbar discectomy involving 42 patients concluded that dexamethasone injection after incision decreased morphine use in the immediate postoperative course (5).

When compared to the group of patients getting saline, we discovered that the group of patients receiving 8 mg of dexamethasone consumed much less paracetamol throughout the first 24 hours. Fujii et al (16) performed a prospective, randomized, double-blind, controlled trial to determine whether dexamethasone is effective at lowering the need for analgesics after middle ear surgery. Individuals who got dexamethasone 8 mg had less need for indomethacin—an anti-inflammatory drug—to manage their postoperative pain than patients who received a placebo. Our findings indicate that intravenous dexamethasone is efficient in reducing postoperative pain and the need for analgesics during the first 24 hours after surgery, in patients who had undergone middle ear surgery.

## Limitations

Our study has some limitations. The design of this study did not compare one analgesic to another, to start. Furthermore, different operators were involved in this study. The last point is that we didn’t evaluate chronic pain after discharge from the hospital.

## Conclusion

 Intravenous dexamethasone is efficient in reducing postoperative pain and analgesic needs in patients having middle ear surgery within the first 24 hours.

## References

[1] Wittekindt D, Wittekindt C, Meissner W ( 2012). Guntinas-Lichius O [Postoperative pain assessment after middle ear surgery. HNO.

[2] Afman CE, Welge JA, Steward DL (févr 2006). Steroids for post-tonsillectomy pain reduction: meta-analysis of randomized controlled trials. Otolaryngol Head Neck Surg.

[3] Fujii Y, Nakayama M (juin 2009). Dexamethasone for the reduction of postoperative nausea and vomiting and analgesic requirements after middle ear surgery in adult Japanese patients. Methods Find Exp Clin Pharmacol.

[4] De Oliveira GS, Almeida MD, Benzon HT, McCarthy RJ ( 2011). Perioperative single dose systemic dexamethasone for postoperative pain: a meta-analysis of randomized controlled trials. Anesthesiology.

[5] Effect of high-dose intravenous dexamethasone on postlumbar discectomy pain.

[6] Liu K, Hsu CC, Chia YY ( 1998). Effect of dexamethasone on postoperative emesis and pain. Br J Anaesth.

[7] Waldron NH, Jones CA, Gan TJ, Allen TK, Habib AS ( 2013). Impact of perioperative dexamethasone on postoperative analgesia and side-effects: systematic review and meta-analysis. Br J Anaesth.

[8] Wang JJ, Ho ST, Liu YH, Lee SC, Liu YC, Liao YC ( 1999). Dexamethasone reduces nausea and vomiting after laparoscopic cholecystectomy. Br J Anaesth.

[9] Roach JT, Sufka KJ ( 2003). Characterization of the chick carrageenan response. Brain Res.

[10] Methylprednisolone and ketorolac rapidly reduce hyperalgesia around a skin burn injury and increase pressure pain thresholds.

[11] Xie W, Luo S, Xuan H, Chou C, Song G, Lv R (2006). Betamethasone affects cerebral expressions of NF-kappaB and cytokines that correlate with pain behavior in a rat model of neuropathy. Ann Clin Lab Sci.

[12] Role of lipocortin-1 in the anti-hyperalgesic actions of dexamethasone.

[13] Barnes PJ ( 2005). Molecular Mechanisms and Cellular Effects of Glucocorticosteroids. Immunology and Allergy Clinics of North America.

[14] De Oliveira GS, Almeida MD, Benzon HT, McCarthy RJ ( 2011). Perioperative single dose systemic dexamethasone for postoperative pain: a meta-analysis of randomized controlled trials. Anesthesiology.

[15] Ahn JH, Kim MR, Kim KH ( 2005). Effect of dexamethasone on postoperative dizziness, nausea and pain during canal wall-up mastoidectomy. Acta Otolaryngol.

[16] Fujii Y, Nakayama M ( 2009). Dexamethasone for the reduction of postoperative nausea and vomiting and analgesic requirements after middle ear surgery in adult Japanese patients. Methods Find Exp Clin Pharmacol.

